# User-Centered Design and Evaluation of a Web-Based Decision Aid for Older Adults Living With Mild Cognitive Impairment and Their Health Care Providers: Mixed Methods Study

**DOI:** 10.2196/17406

**Published:** 2020-08-19

**Authors:** Laura-Mihaela Bogza, Cassandra Patry-Lebeau, Elina Farmanova, Holly O Witteman, Jacobi Elliott, Paul Stolee, Carol Hudon, Anik M C Giguere

**Affiliations:** 1 Laval University Research Centre on Community-Based Primary Health Care Quebec, QC Canada; 2 Quebec Centre for Excellence on Aging Québec, QC Canada; 3 Department of Family Medicine and Emergency Medicine Laval University Québec, QC Canada; 4 Institute of Health Policy, Management and Evaluation University of Toronto Toronto, ON Canada; 5 School of Public Health and Health Systems University of Waterloo Waterloo, ON Canada; 6 School of Psychology Laval University Québec, QC Canada

**Keywords:** decision aid, mild cognitive impairment, elderly, decision support technique, aging

## Abstract

**Background:**

Mild cognitive impairment (MCI) is often considered a transitional state between normal and pathologic (eg, dementia) cognitive aging. Although its prognosis varies largely, the diagnosis carries the risk of causing uncertainty and overtreatment of older adults with MCI who may never progress to dementia. Decision aids help people become better informed and more involved in decision making by providing evidence-based information about options and possible outcomes and by assisting them in clarifying their personal values in relation to the decision to be made.

**Objective:**

This study aimed to incorporate features that best support values clarification and adjust the level of detail of a web-based decision aid for individuals with MCI.

**Methods:**

We conducted a rapid review to identify options to maintain or improve cognitive functions in individuals with MCI. The evidence was structured into a novel web-based decision aid designed in collaboration with digital specialists and graphic designers. Qualitative and user-centered evaluations were used to draw on users’ knowledge, clarify values, and inform potential adoption in routine clinical practice. We invited clinicians, older adults with MCI, and their caregivers to evaluate the decision aid in 6 consecutive rounds, with new participants in each round. Quantitative data were collected using the Values Clarity and Informed subscales of the Decisional Conflict Scale, the System Usability Scale, the Ottawa Acceptability questionnaire, and a 5-point satisfaction rating scale. We verified their comprehension using a teach-back method and recorded usability issues. We recorded the audio and computer screen during the session. An inductive thematic qualitative analysis approach was used to identify and describe the issues that arose. After each round, an expert panel met to prioritize and find solutions to mitigate the issues. An integrated analysis was conducted to confirm our choices.

**Results:**

A total of 7 clinicians (social workers, nurses, family physicians, psychologists) and 12 older (≥60 years) community-dwelling individuals with MCI, half of them women, with education levels going from none to university diploma, were recruited and completed testing. The thematic analysis revealed 3 major issues. First, the user should be guided through the decision-making process by tailoring the presentation of options to users’ priorities using the values clarification exercise. Second, its content should be simple, but not simplistic, notably by using information layering, plain language, and pictograms. Third, the interface should be intuitive and user friendly, utilize pop-up windows and information tips, avoid drop-down menus, and limit the need to scroll down. The quantitative assessments corroborated the qualitative findings.

**Conclusions:**

This project resulted in a promising web-based decision aid that can support decision making for MCI intervention, based on the personal values and preferences of the users. Further ongoing research will allow its implementation to be tested in clinical settings.

## Introduction

### Mild Cognitive Impairment

Mild cognitive impairment (MCI) is characterized by a decline in cognitive functioning that is more pronounced than normal aging but does not significantly compromise activities of daily living. The impairment can affect memory, language, problem solving, or attention, among others [1]. MCI is common in adults aged ≥60 years, with a prevalence of 6.7% among adults aged 60 to 64 years and 25.2% among adults aged 80 to 84 years [2]. MCI is often considered an early manifestation of pathologic cognitive decline, such as dementia [3]. However, as it can have several causes, its prognosis varies considerably and, although there is a clear risk of dementia, it is also possible to see stability and improvement over time [4]. A diagnosis of MCI therefore carries uncertainty and the risk of overtreatment of older adults with MCI who may never progress to dementia [4]. When worried individuals consult their physicians, they need clear information on the options available to prevent cognitive decline and on each option’s probable benefits and harms.

### Decision Aids

Decision aids are standardized tools that provide this information and support decision making for the best course of action [5,6]. They help people become more informed and engaged in decision making by providing evidence-based information about all available options and their positive and negative outcomes, and by assisting them in clarifying their personal values in relation to the decisions to be made [7]. These tools help improve people's knowledge regarding options and risks, increase decisional comfort, stimulate them to participate in decision making, and improve patient-professional communication when compared with care without the use of decision aids [7]. In recent years, our team has studied the design features of printable decision aids, or *decision boxes*, to help health care professionals (HCPs) and patients understand evidence-based information and promote shared decision making [8-10]. However, little is known about how detailed decision aids need to be to support an evidence-informed and value-based decision [7]. Moreover, decision aids have seldom been tested with older populations [11] or in the context of MCI.

Considering the differences between the decision-making patterns of older and younger adults [12], such as the preference of older adults for fewer options than younger adults [13], lower levels of literacy and numeracy among older adults compared with younger adults [14,15], the presence of caregivers in the decision-making circles of older adults [16,17], and more frequent sensory deficits among older adults compared with younger adults [18], there is a need for a decision aid developed in a user-centered way with input from older adults, family or friend caregivers, and professionals, to empower and support this specific population in health care decision making.

### Design of Web-Based Decision Aids for Older Adults With Mild Cognitive Impairment

With the internet becoming a primary source of information for consumers looking for health information and advice [19,20], web-based decision aids need to be investigated further [21]. Although several models of web-based decision aids have been tested, evidence about the best use of interactive features is still limited. A 2016 systematic review of studies conducted with adults found that the use of interactive features in web-based decision aids can help improve decision making in preference-sensitive contexts [22]. The review concluded that features that allowed users to control the order in which they viewed the content, its level of detail, and the type of evidence presented were helpful in supporting decision making. However, it also demonstrated that having too many tailoring options might cause more decisional conflict and decreased knowledge compared with decision aids without these features. The review also concluded that features such as values clarification exercises, feedback, and social support had varying effects on decision making, depending on their design, which points to a need for further studies.

Therefore, this study aimed to identify and apply features that best support values clarification and understanding of evidence in older adults with MCI, and to adjust the level of detail of a web-based decision aid inspired by the decision box template, for individuals with MCI.

## Methods

### Study Approach and Design

We used a user-centered approach [23] to design a web-based decision aid that improves the users’ knowledge, values clarification, and its adoption in routine clinical practice. We asked individuals with MCI, as well as HCPs, to evaluate the web-based decision aid in 6 consecutive rounds using mixed methods comprising semistructured interviews and questionnaires [24-26]. New participants were recruited for each round. We also verified the comprehension of the information using a teach-back method and recorded any usability issues. We recorded the audio and computer screen during the session. After each evaluation round, we analyzed the data and, based on the findings, tailored the web-based decision aid to improve user experience.

### Prototype Conception

On the basis of a published rapid-review approach [27], we synthesized evidence on the benefits and harms of the available options to maintain or improve cognitive functions in older adults with MCI. The evidence gathered was structured into a decision box template, which is in accordance with the international patient decision aid standards and has been described in earlier publications [8,9]. To ensure that the content addressed the users’ needs and was scientifically valid, we then formed an expert advisory panel consisting of 8 clinicians (2 nurses, 2 family physicians, a neuropsychologist, a geriatrician, an occupational therapist, and a pharmacist). We invited the members of the panel to review the content, which was then adjusted to take into account their recommendations before testing with potential users.

The informational content was then inserted into a mock-up designed to support older adults with MCI in making informed and value-based decisions with their health care team. More specifically, we designed the web-based decision aid to (1) allow people to weigh the probabilities of experiencing benefits against the probabilities of experiencing harms, for all the available options; (2) allow people to clarify what is most important to them; and (3) support people in voicing their priorities to their health care team. The design process was inspired by steps 3 to 5 of the Center for eHealth and Wellbeing Research guidelines [28,29], which describe the design, operationalization, and evaluation of a tool. Development was initially informed by a review of the literature in the process of developing web-based decision aids and their features [22,30-32] and select examples of web-based evidence summaries and decision aids (eg, drug facts boxes developed by Steven Woloshin and Lisa Schwartz and used by the US Food and Drug Administration [no longer available in a web-based format], MAGICapp developed by Making Grading of Recommendations Assessment, Development, and Evaluation [GRADE] the Irresistible Choice, and option grids developed by Peter Scalia, Glyn Elwyn and Marie-Anne Durand and commercialized by EBSCO Health). Then, in a collaborative and iterative effort, we conducted several brainstorming sessions to discuss the functionality, user experience, and purpose of each of the web-based decision aid’s components. The team comprised a computer analyst, 2 graphic designers specialized in website interfaces (one of whom was EB), a researcher specialized in dementia and health literacy (EF), and the principal investigator (AG), who specializes in shared decision making and is the developer of the decision box template.

**Figure 1:**
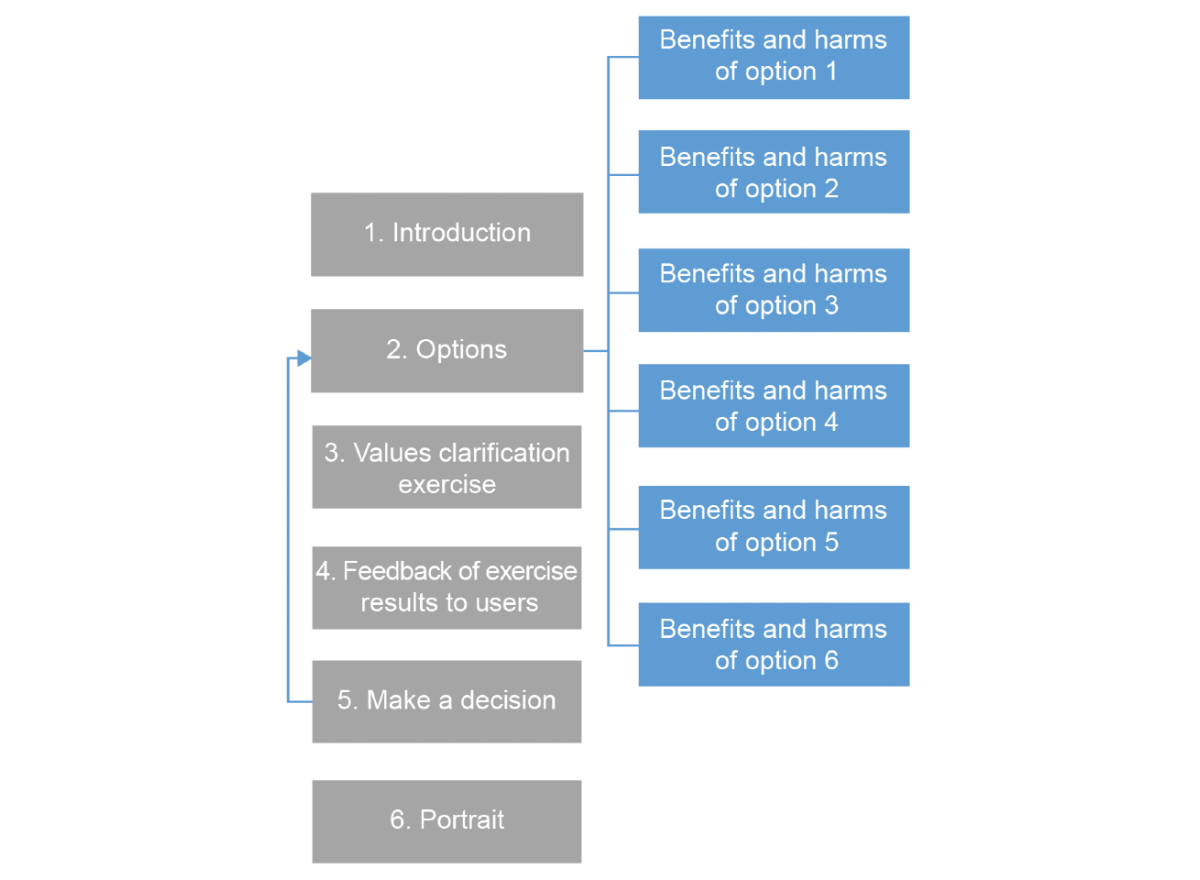
Navigation through the various sections of the web-based decision box as planned in the first prototype, before user testing.

In this prototype, potential users were guided through a linear decision-making process going from a general introduction to a description of the available options to a values clarification exercise before making a decision (Figure 1). Users could then go back to change their choices as many times as they liked. Once a decision was made, they could view their profile, which summarized their priorities, decisions, and decisional comfort.

### Population and Sampling Strategy

Adults aged ≥60 years with MCI, their informal caregivers if they had one, and HCPs of any profession who practiced in family medicine clinics were eligible to participate. We initially invited HCPs who worked in 7 primary care clinics in the areas surrounding Quebec City. We sent them an email to explain the study and invited them to confirm their interest in participating in the study using a link to a web survey. We then asked those who agreed to participate to identify eligible individuals among their patients, who scored between 18 and 26 on the Montreal Cognitive Assessment [33] and to contact them to ask their permission for the research team to contact them. Only the contact details of people who expressed an interest in participating in the study were shared with the research team. As we experienced challenges recruiting participants with this strategy, we also invited people with MCI who were registered in a Quebec database managed by one of us (CH). A diagnostic procedure described elsewhere [34] was used to verify that these participants with MCI met the Petersen criteria for single- or multiple-domain MCI [35], which include (1) a subjective memory complaint, corroborated by an informant; (2) an objective memory impairment, based on a cut-off score of ≥1.5 SD below age-adjusted norms on at least one standardized memory test in the neuropsychological battery; (3) preserved general cognitive functioning; (4) largely intact functional activities; and (5) not demented.

### Evaluations

We invited the participants to evaluate the web-based decision aid during a 60-min session, conducted either in participating clinics, the research center, or in their own homes. The duration of the session was adapted to the capacities of each participant.

At the beginning of the session (pretest), all participants completed a self-report questionnaire to record their sociodemographic characteristics, and older adults completed the *Values Clarity* and *Informed* subscales of the Decisional Conflict Scale (DCS) to rate, respectively, their feeling of being clear about personal values for benefits and risks/side effects, and their feelings of being informed [36]. We reversed the DCS subscale scores to facilitate interpretation; increased scores represented increases in feelings of being clear about personal values and being informed. The research assistant was available to support the participants in completing the questionnaire as needed.

Then, they reviewed the web-based decision aid during a semistructured interview that used a think-aloud approach [37]. A trained interviewer followed an interview guide designed to evaluate the participants’ information needs and their perception of the strengths and limitations of the web-based decision aid. The guide used to interview older adults also contained questions inspired by the *teach-back* method to assess the users’ understanding of the information. Teach-back is used by HCPs and consists of asking patients to explain the information back to them [24,38-41]. The discussions were audio recorded, and we captured the participants’ navigation on their computer screens using the video feature.

At the end of the think-aloud evaluation of the web-based decision aid (posttest), all participants completed 5 items of the Ottawa Acceptability questionnaire [42] to assess the quality of decision aids as well as the 10-item System Usability Scale (SUS) [43,44]. They also rated their satisfaction with the web-based decision aid using a single item, 5-point smiley face rating scale. In addition, the HCPs completed a number of questions to explore their willingness to adopt the decision aid in clinical settings [25]. At posttest, the participating older adults once again completed the *Values Clarity* and *Informed* subscales of the DCS.

### Tailoring the Web-Based Decision Aid to Users’ Needs

After each round, a thematic qualitative analysis of the discussions highlighted the strengths and limitations of the web-based decision aid. One researcher (LB, EF, or CP) conducted the analysis, and another reviewed it (AG). The expert panel then met to prioritize the issues and find solutions to mitigate them. To this end, members of the same interdisciplinary expert panel who created the prototype met to review the qualitative findings. The experts started by prioritizing each issue using the following severity scale [45]: (1) critical flaw: if unresolved, then users will not be able to complete the task; (2) serious flaw: several users may be frustrated and give up; (3) minor flaws: users may be annoyed, but they will complete the task. They then chose solutions by considering the magnitude, frequency, and severity of these problems, and how they prevented the web-based decision aid from achieving its primary goal (supporting evidence-informed and value-based decision making).

The same evaluation and tailoring process was then used again in 5 more rounds, with new participant subsamples each time.

### Integrated Analyses

In the final analysis, we integrated the findings from the 6 rounds of qualitative and quantitative evaluations into the web-based decision aid design. This triangulation process served to validate our findings [26,46,47] and to form a comprehensive picture of the web-based decision aid’s acceptability and usability and of the level of detail required to support evidence-informed and value-based decision making by older adults with MCI, their caregivers, and HCPs. We determined the level of detail based on the participants’ knowledge and action. An appropriate level of detail was interpreted from the decision aid’s potential to inform decision making and increase decisional comfort, thereby inciting the user to take action. More specifically, we considered the level of detail as appropriate if the decision aid increased decisional comfort (ie, achieved *100* or a value closer to *100* on the DCS informed subscale at posttest) and if the participants demonstrated high levels of intention to use the decision aid to make a decision following the diagnosis of MCI.

We assessed the suitability of the values clarification exercise using the participants’ responses to the DCS *Values Clarity* subscale and to open-ended questions about their experience with the values clarification exercise. We considered the values clarification exercise as suitable if it increased the participants’ feelings of being clear about personal values (ie, achieved *100* on the DCS *Values Clarity* subscale) and if participants (especially those in the fifth and sixth evaluation rounds) reported a positive experience using the decision aid.

### Ethical Considerations

Ethical approval to conduct this pilot study was granted by the Integrated University Health and Social Services Center of Québec City (*CIUSSS-Capitale-Nationale*, no. 2016-2017-08).

## Results

### Participant Characteristics

We recruited 7 HCPs (3 social workers, 2 nurses, 1 physician, and 1 psychologist; Table 1) and 12 community-based older adults with MCI (Table 2). Out of the 12 older adults, 3 did the interview in the presence of a friend or family member, whom they did not consider to be a caregiver, and 9 did it alone with the research assistant. Of which, 2 of the persons with MCI were aged between 60 and 64 years, 6 between 65 and 74 years, 3 between 75 and 84 years, and 1 was >85 years. The participants’ education levels varied from none to university degree.

**Table 1:** Description of the characteristics of the participating health care professionals (N=7).

Characteristic		Values, n (%)
**Age (years)**		
	<30	1 (14)
	30-39	4 (57)
	50-59	2 (29)
**Sex**		
	Female	6 (86)
	Male	1 (14)
**Profession**		
	Physician	1 (14)
	Nurse	2 (29)
	Social worker	3 (43)
	Psychologist	1 (14)
**Practice experience (years)**		
	0-4	2 (29)
	5-9	0 (0)
	10-14	1 (14)
	15-19	2 (29)
	20-24	1 (14)
	25-29	0 (0)
	30-34	0 (0)
	35-39	1 (14)

**Table 2:** Description of the characteristics of the participating older adults with mild cognitive impairment (N=12).

Characteristic		Values, n (%)
**Age (years)**		
	60-64	2 (17)
	65-74	6 (50)
	75-84	3 (25)
	≥85	1 (8)
**Sex**		
	Female	6 (50)
	Male	6 (50)
**Ethnicity**		
	White	12 (100)
**Primary language**		
	French	12 (100)
**Marital status**		
	Single	1 (8)
	Married or common-law relationship	9 (75)
	Widow	2 (17)
**Education** ^a^		
	No formal education	1 (8)
	High school diploma	4 (33)
	College diploma	3 (25)
	University diploma	3 (25)
**Place of residence**		
	Home	12 (100)
**Has a caregiver**		
	Yes	2 (17)
**If so, who?**		
	Family member	1 (8)
	Friend	1 (8)
**Lives with their caregiver**		
	Yes	1 (8)
	No	1 (8)

^a^One of the participants had missing data for education.

We conducted 6 evaluation rounds, starting with the prototype version (V1), and then produced 5 different versions (V2-V6) before creating the final version (VF) that is currently available on the Decision Box website [48]. Of which 6 HCPs evaluated the prototype (V1) and 1 evaluated V2. Additionally, 3, 3, 2, 3, and 1 older adult with MCI evaluated versions 2, 3, 4, 5, and 6, respectively. The participating HCPs reviewed only the first and second mock-up.

### Qualitative Findings

A total of 3 major issues emerged from the qualitative analysis: (1) users should be guided through the decision-making process; (2) the content should be simple, but not simplistic; and (3) the interface should be intuitive and user friendly.

#### Guided Decision-Making Process

As mentioned, the first prototype (V1) proposed a linear directional navigation, going from general to specific information based on users’ preferences (Figure 1). It led users to read all available options before clarifying their values and making a decision. As a result, we observed that participants often paused in the *Benefits and Harms* section and did not complete the next steps because they grew tired or lost focus:

Participant: Geez, this is quite something, isn’t it? You’re making me work hard, you know. Where are we at now? [interviewer explains the next steps]. I don’t know whether I’m coming or going.

Older adult #1001

Interviewer: What do you mean?

Participant: Well, I...I’m tired, now.

Hence, to ensure participants would complete the values clarification exercise, we used a nondirectional navigation strategy in V2, allowing users to start either with a review of the options or with the values clarification exercise. This also helped meet the needs of HCPs who visit the web-based decision aid without their patients, to review the evidence about the available options. However, user testing revealed that this nondirectional design confused several users who wondered about the *correct* path:

[After the interviewer explains that they can choose whichever path they prefer] So, are we going to explore all the options or uhh…?

Nurse #3009, V2

**Figure 2:**
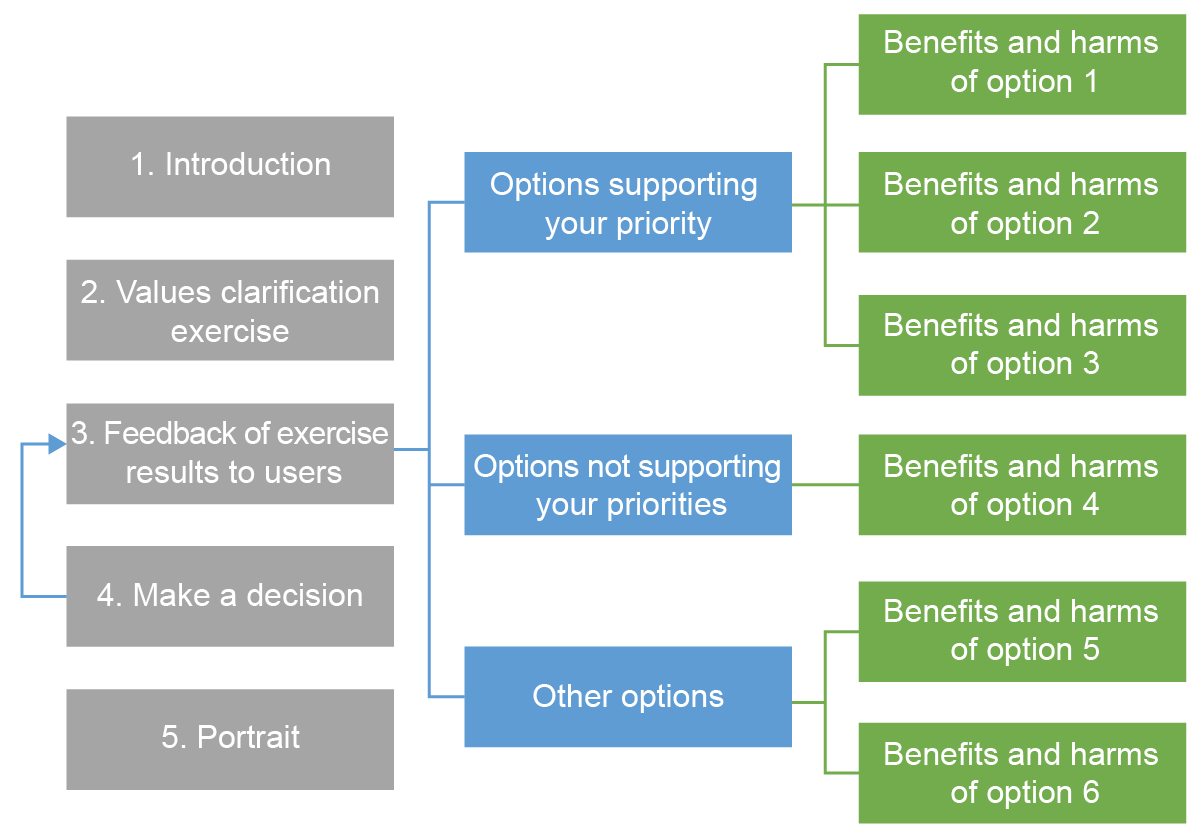
Navigation through various sections of the web-based decision box as planned after user testing.

The experts therefore decided to revert to a directional navigation in the next version (V3), leading users to complete the values clarification exercise before reviewing the options (Figure 2), which is in the opposite direction from V1. Once people had clarified their values, the web-based decision aid presented them with the options that matched their values. The other options were still available for review in case they wished to learn more about them.

Moreover, V3 included clear indications of the next steps to improve usability. Despite directional navigation, the prototype still allowed users to access any page at any time, using a progression bar that we added above the menu.

After V3, the users did not report feeling fatigued, overworked, or lost as they went through the web-based decision aid. This was likely because they could read the options most relevant to them and then move on without feeling that they had missed out. This meant that they could better manage their navigation to match their energy levels and improve their likelihood of making it through all the decision-making steps. Furthermore, it helped simplify the web-based decision aid, as discussed in detail in the following section because it limited the number of options to be read before making a decision. This modification also made the web-based decision aid more patient centered, as opposed to professional centered.

#### Simple but Not Simplistic

Throughout the various versions, participants consistently asked for a simpler design. Therefore, the challenge was to simplify the web-based decision aid while retaining its informative value to ensure that users would have enough information to make informed and value-based decisions. The amount of information to read (content density) and its complexity (content clarity) were of major concern to the users.

##### Content Density

Participants made frequent comments about the overwhelming nature of the information, especially in the *Introduction* and *Options* sections of V1 as shown in Multimedia Appendix 1 (Warning: this is a preliminary version and the data is not final. For a final version, please see [48].). In V1, we observed that many participants did not read all the available information in these sections:

Oh my! It’s going to take so long to read all that.

Social worker #3004, V1

To address these concerns, we modified the information layout in the *Introduction* section. The information that was initially structured into columns was put into bulleted lists, in separate accordion tabs that could be expanded by clicking on a *+* button. In V3, we also removed the references from the text and moved them to a separate page, as shown in Multimedia Appendix 2.

To improve the *Options* section, we tried integrating the options to the values clarification exercise results page. This was meant to allow people to read the information most important to them first when they had the most focus. This strategy offered a more straightforward reading path, but some participants still did not read the information regarding all the options in V4:

Wow, there are a lot of things [talking about all the options]! I won’t read all that. [Older adult #1008, V4]

After 5 unsuccessful versions trying to limit the users’ burden as they read the options, we classified the options into 3 subcategories: (1) options that matched their priority, (2) options that did not match their priority, and (3) other options to consider. People could only view the subcategories related to a single priority at a time. This prevented us from having to remove information that we deemed essential to support informed, value-based decision making (which would have led to a simplistic design), while still allowing us to highlight the information most relevant to the user.

##### Content Clarity

###### Scientific Terms

Several of the HCPs who evaluated the first version found the content too scientific and potentially difficult to understand for laypeople, as pointed out by this participant:

I mean, maybe a doctor or a nurse will be able to understand, but me, I don’t have… I don’t have any medical training. I’m a social worker, so when you show me terms like that,… you lose me.

Social worker #3004, V1

Users did not like the style either, as pointed out by this participant:

It’s very scientific. I don’t identify with the text.

Older adult #1008, V4

The content was progressively modified to use more lay terms, as demonstrated by the change of the decision aid title from Mild Cognitive Impairment to Mild Problems with Thinking and Memory in the final version.

###### Probabilities

**Figure 3:**
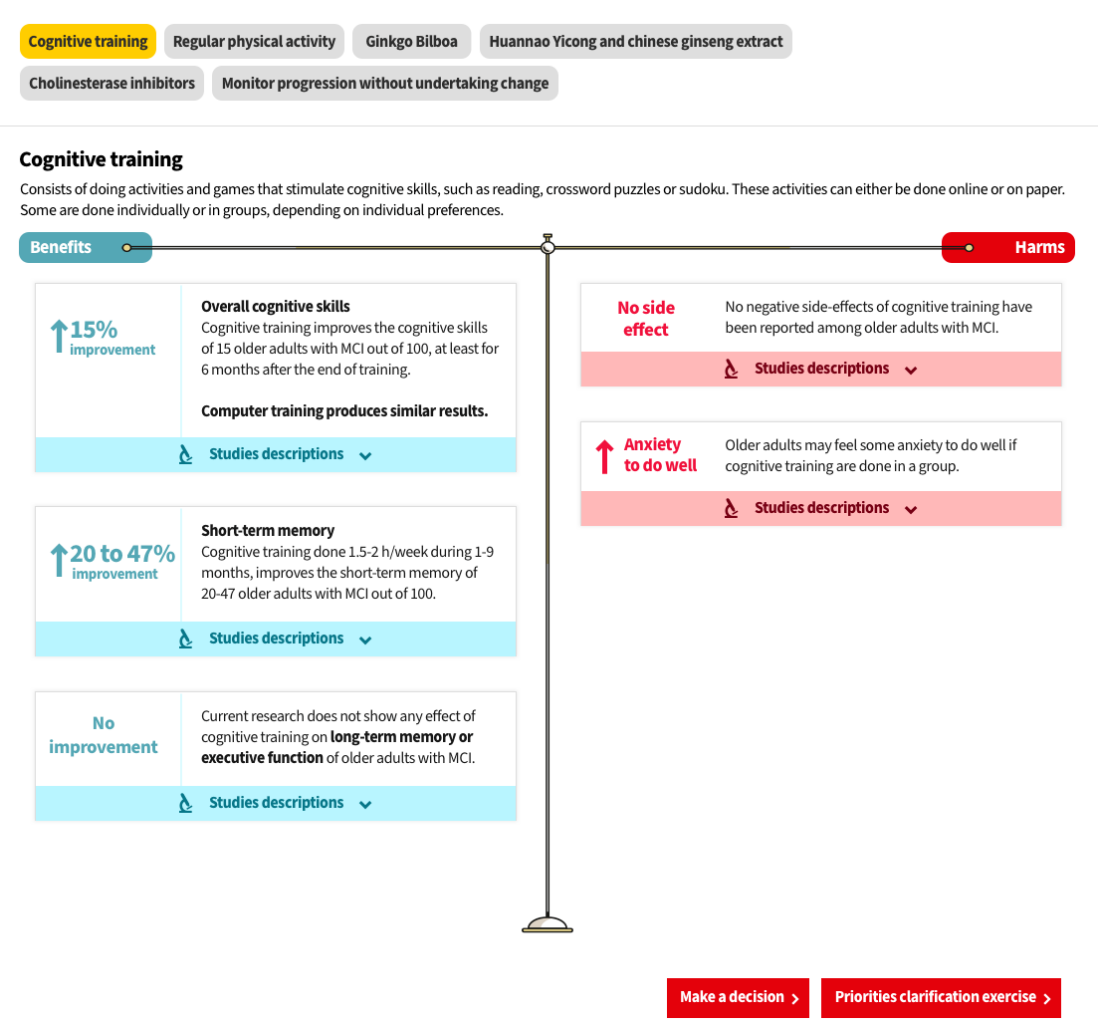
Presentation of one of the options in prototype V2. Warning: This is a preliminary version, and the data are not final.

Information clarity was also jeopardized by the users’ misunderstanding of the probabilities presented in the *Options* section (V2, V3, V4, and V6). In fact, most participants misunderstood the absolute effect sizes presented in larger font sizes, in percentages, in V1 (Figure 3; warning: this is a preliminary version and the data is not final. For a final version, please see [48]). They interpreted them as mean effect sizes (eg, a 15% increase in mental capacities) while in reality they represent the proportion of people who might benefit from the intervention (15 persons out of 100 improve their mental capacities):

It can improve by 11% compared to my… compared to now.

Older adult #1004, V3

**Figure 4:**
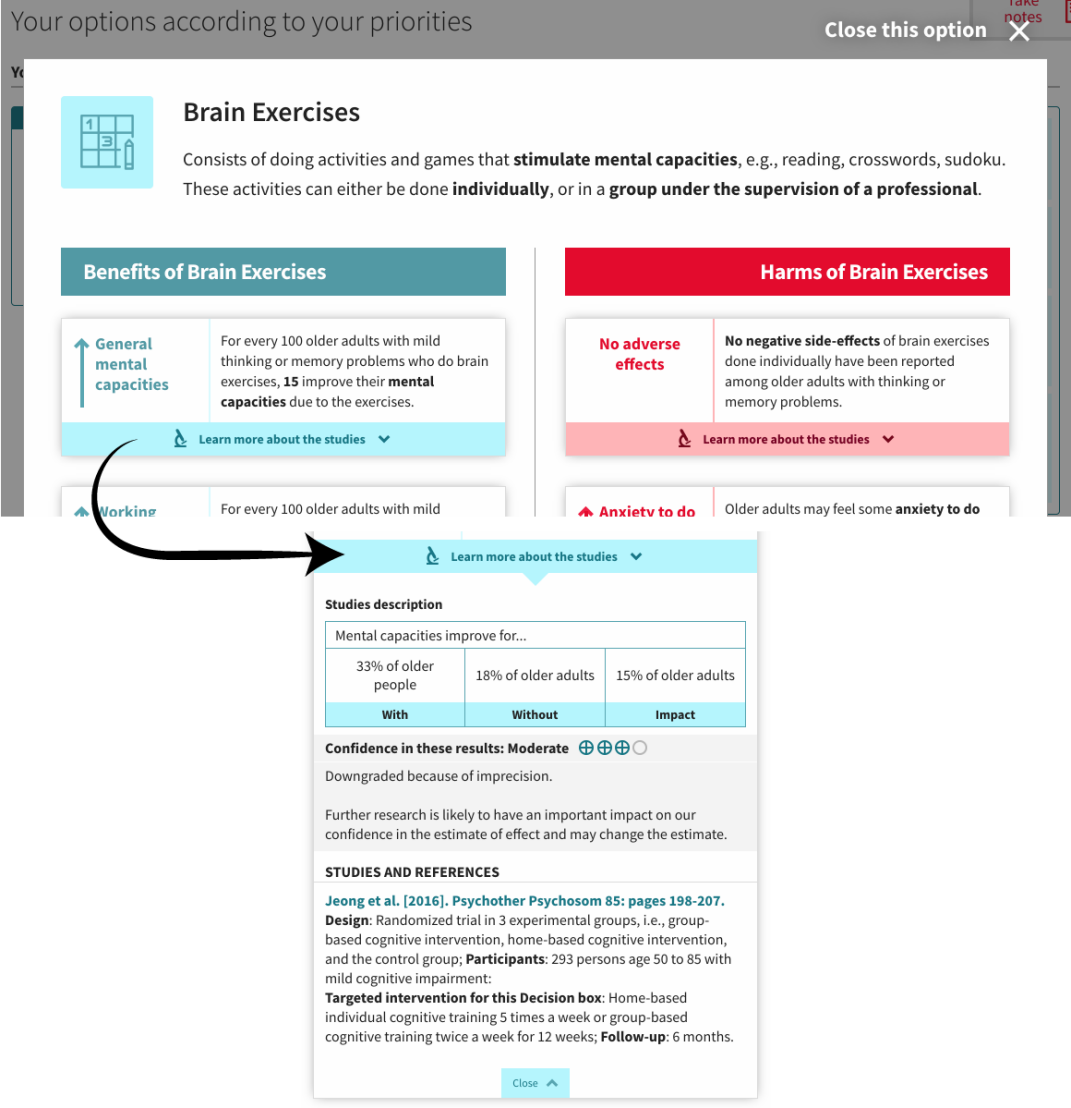
Presentation of one of the options in the final version showing the pop-up format and with an enlargement showing an example of the expanded accordion tabs to learn more about the grading of recommendations assessment, development, and evaluation ratings.

To minimize influence on decision making, we replaced numbers and percentages with text in the final version. For example, we replaced “↑15% improvement” with “↑ General mental capacities” in a slightly larger font size (Figure 4), and we presented the detailed proportions (*for every 100 older adults, 15 improve their mental capacities*) in smaller font on the right-hand side.

###### Grading of Recommendations Assessment, Development, and Evaluation Ratings on Confidence in the Results

Participants also often misunderstood the GRADE ratings associated with each effect size in the earlier versions of the web-based decision aid (V1 and V4):

Oh, this is high: 4 out of 4 stars! It’s like when you choose a holiday spot down south!

Older adult #1007, V4

Therefore, we layered the GRADE ratings, allowing easy access by the most curious users under a *Learn more about the studies* accordion tab placed under each option in V5 of the web-based decision aid (Figure 4).

##### Feedback After the Values Clarification Exercise

**Figure 5:**
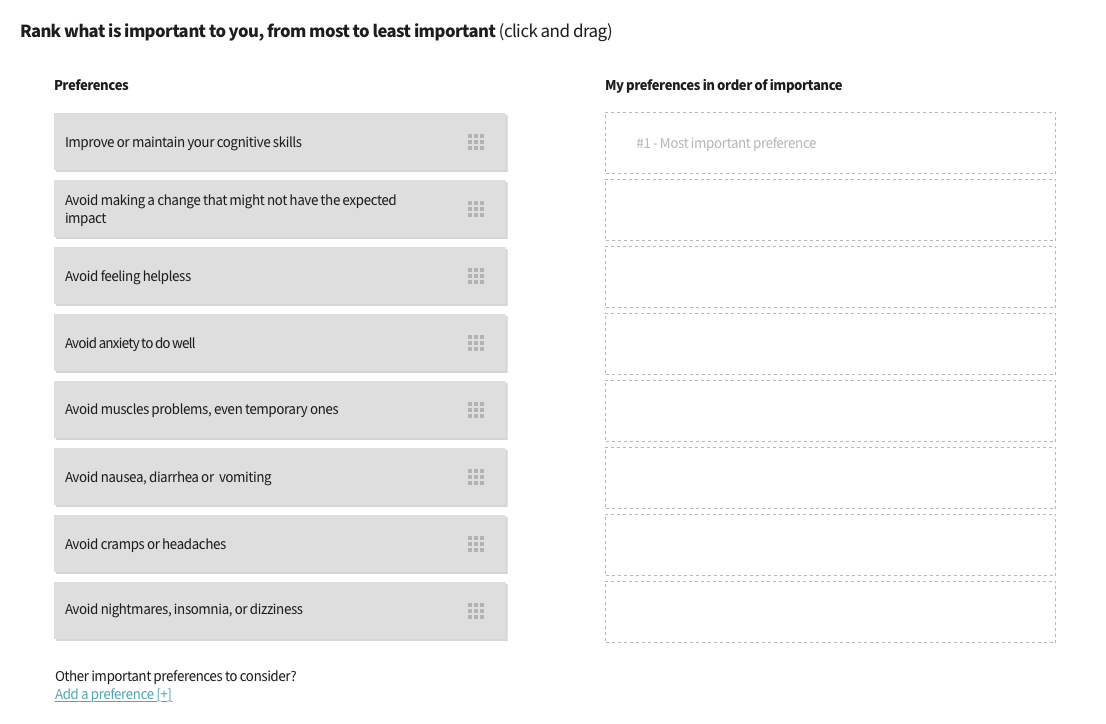
First prototype version of the web-based decision aid, showing the values clarification exercise.

**Figure 6:**
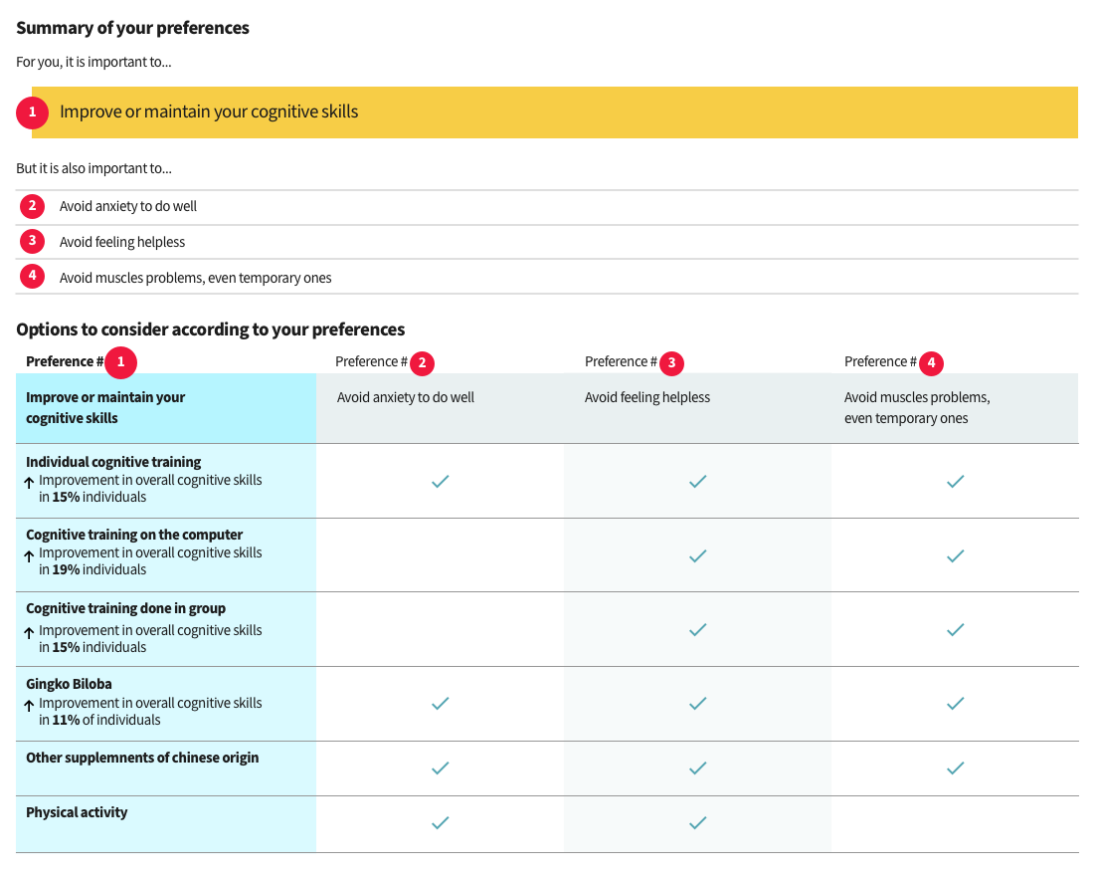
First prototype version of the web-based decision aid, showing the feedback page after the exercise. Warning: This is a preliminary version, and the data are not final.

As mentioned earlier, the feedback provided to users after they had completed the values clarification exercise (Figure 5) required several modification rounds before it was understood. The goal was to present the options that matched the person’s values. The options were initially shown in a matrix table, which most users misunderstood (Figure 6; warning: this is a preliminary version and the data is not final. For a final version, please see [48]), as demonstrated by this participant’s comments:

Interviewer: Did you think the check marks were choices you had to make?

Participant: Yeah, yeah...I thought I was the one placing the check marks.

Older adult #1006, V3

The modifications made to the matrix table in the next round did not improve the participants’ understanding, as demonstrated by this quote:

Here, what am I supposed to do? [...] I have to make a check mark...Should I make a check mark? What is it? [...] I see there’s an X. If I click, will it give me a check mark?

Older adult #1007, V4

**Figure 7:**
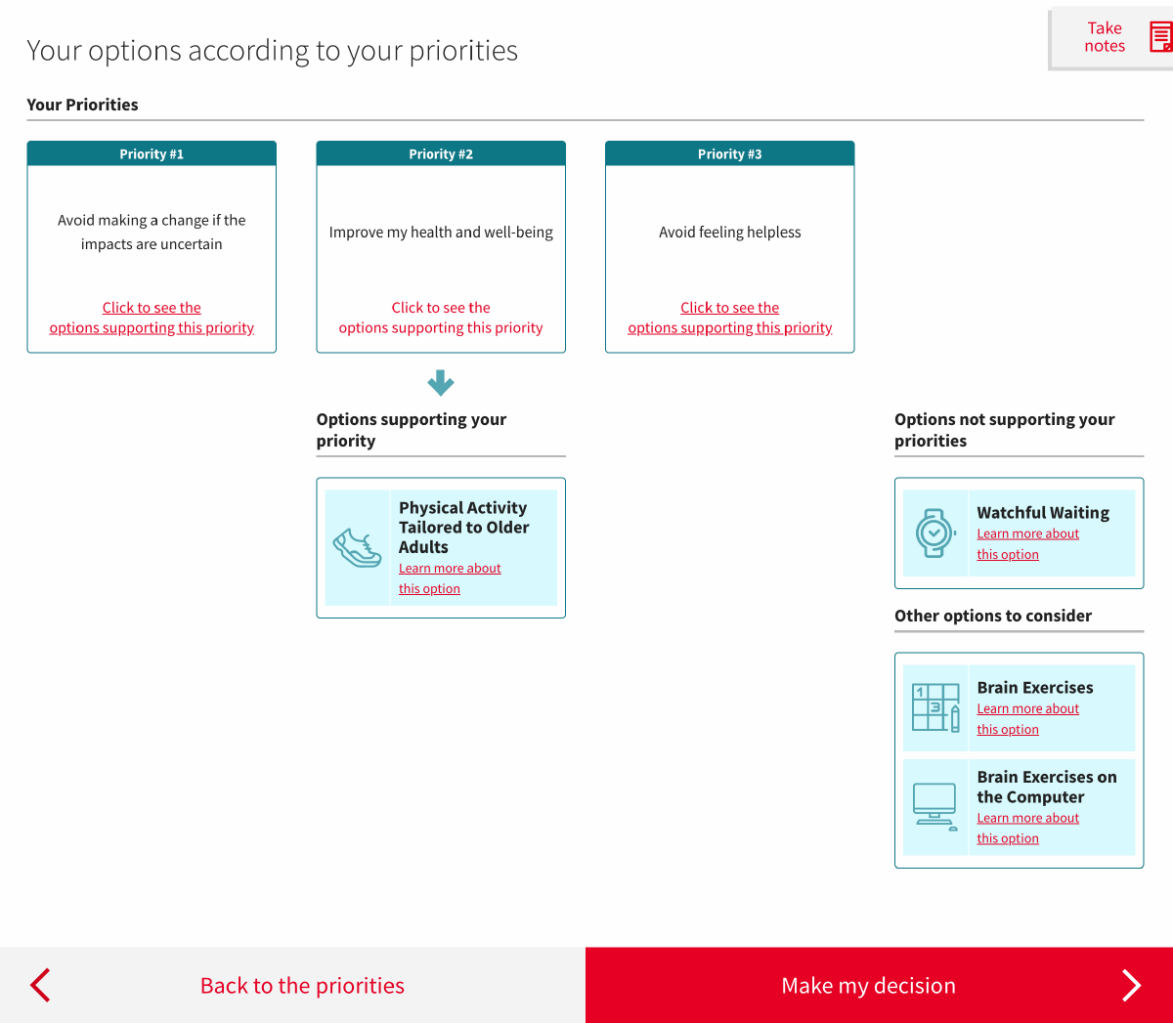
Final version of the web-based decision aid, showing the feedback page after the exercise.

**Figure 8:**
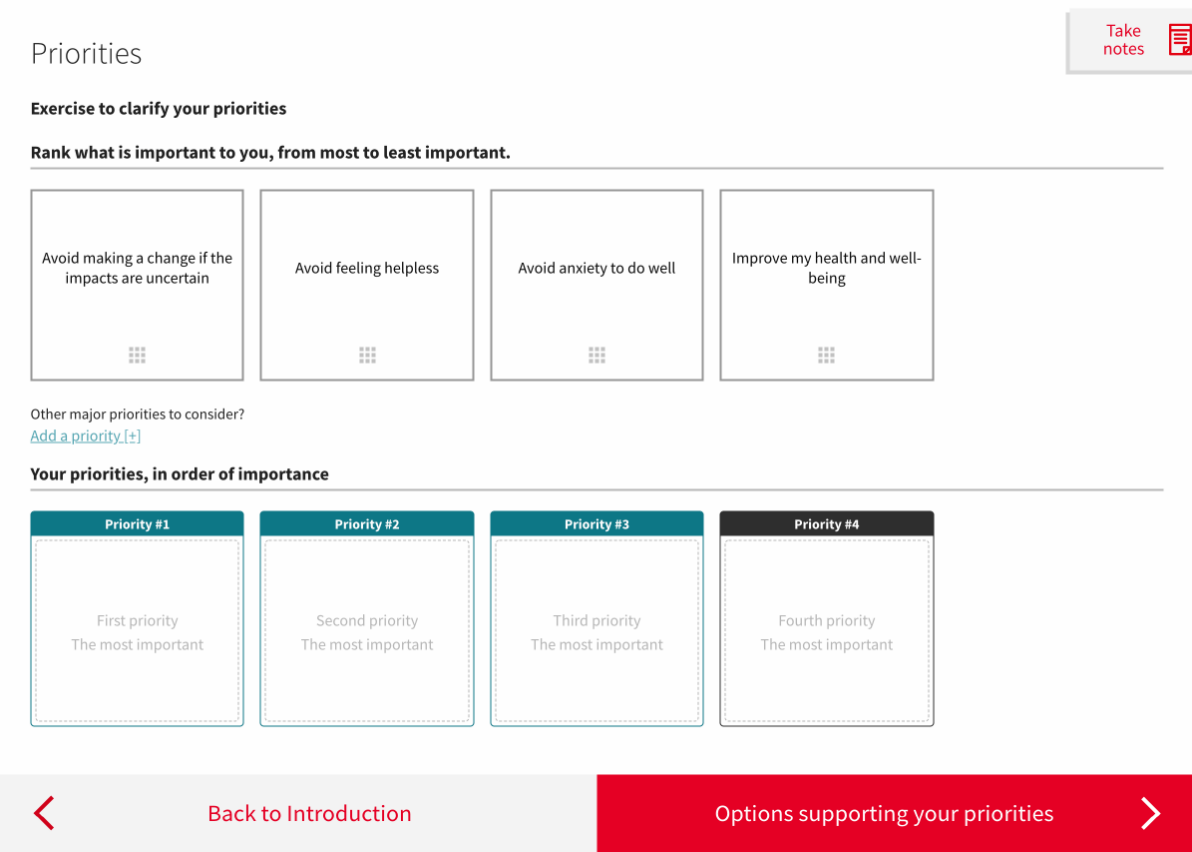
Final version of the web-based decision aid, showing the values clarification exercise.

Therefore, we decided to limit the feedback to its simplest form. We discarded the matrix table in V5, and instead listed the options as a series of boxes underneath each of the values (described hereafter as *priorities*) selected by the person, with a maximum of 3 values (Figure 7). We kept a visual connection between the values clarification exercise (Figure 8) and the feedback by using the same format in both pages for the values. We also reduced the number of values presented from 8 to 4, a decision that will be explained later in the *Values Clarification Exercise* section.

We initially included the option of taking cholinesterase inhibitors, as health care providers sometimes consider this medication. However, the scientific evidence described in the decision aid underscored their ineffectiveness in improving cognitive functioning, and this was more confusing than helpful for the participants. Therefore, we removed it from the options available in the subsequent versions. All options lacking evidence of benefits have been removed in the same manner.

##### Pictograms

Once they reached the *Decision* section, several participants did not recall the information related to each of the available options. We, therefore, added a pictogram representing each option every time it was discussed (eg, Figures 4 and 7). This feature was meant to improve recall, and we did indeed observe some improvements in this regard after it was added:

Participant: The definition of...umm of...the sneakers, right?

Older adult #1012, V6

Interviewer: Yes, the physical activity.

Participant: Yes, physical activity.

#### Intuitive and User-Friendly Interface

##### Interface Features

We incorporated several modifications, at all stages, to design an intuitive and user-friendly web-based decision aid. For example, we defined more complex words in a glossary. We initially placed the glossary on a separate page at the end of the web-based decision aid, which was accessible when users clicked on a complex word. However, some users became confused when redirected to a different page; they forgot why they were there and did not know how to get back to the main activity. We therefore arranged for definitions to appear as information tips when the cursor hovers over a word, as shown in Multimedia Appendix 3. This way, users could see the definition of a word while remaining on the *Introduction* page.

We also had to add instructions on how to expand the accordion tabs, as participants did not understand at first (Multimedia Appendix 1):

Participant: [reading out loud] Take into account the patient’s preferences. [to the interviewer] Do I...What do I do now...?

Older adult #1005, V3

Interviewer: Yes, here you could click, it’s a link to open a section with a bit more information about that.

To this end, we added a Learn more link next to the + sign to indicate the possibility of expanding the accordion (Multimedia Appendix 2).

Several users could not find a way to return to the previous page after clicking on an option to read more information about it (Figure 3). Thus, we showed the benefits and harms of the options in a pop-up instead of on a standalone page (Figure 4). This facilitated navigation, as users simply had to close the option to get back to the main page.

We also modified another feature related to the *Decision* section, where users were invited to select an option. We used a drop-down menu in V1 (Multimedia Appendix 4); however, the task was not understood by all participants. We therefore replaced the drop-down menu with checkboxes from V3 onward (Multimedia Appendix 5), which resolved the issue:

Participant: Do we do all that?

Older adult #1001, V2

Interviewer: Have you seen the menu? There is a menu. It says you have to select the option you prefer.

Participant: Oh. My goodness!

Interviewer: If you click on… [interviewer shows the participant how to use the drop-down menu].

##### Values Clarification Exercise

In the first version of the values clarification exercise tested by older adults, most participants needed explanations from the moderator to complete the exercise:

Participant: [Participant reads the priorities out loud] Now you want me to look at that, what do you want to know about what I’m looking at?

Older adult #1002, V2

[Interviewer explains the exercise]

Participant: Uhh, let’s say I put one here, then two...

Caregiver: Actually, aren’t you supposed to move this here?

Interviewer: Exactly.

Several of the participating HCPs felt that there were too many priorities and that the wording was too complex, partly because they were worded negatively. Therefore, we changed the wording and reduced the number of priorities from 8 (Figure 5) to 4 (Figure 8). We also changed the format of the exercise from vertical (Figure 5) to horizontal (Figure 8), primarily to allow the content to fit most computer screens without scrolling down, but also to improve continuity with the feedback, which also displayed the selected priorities horizontally.

#### Quantitative Results

##### Assessments of the Web-Based Decision Aid

The limited number of participants did not allow for any statistical testing to compare the users’ assessment of the web-based decision aid, or impacts of the decision aid, between rounds, or between types of users (Table 3). There is no consistent trend, as the rounds progressed, for any of the studied variables.

##### Impacts of the Web-Based Decision Aid

We did, however, have sufficient data to compare the mean of all users’ ratings on the DCS before and after using the decision aid. We observed statistically significant improvements in older adults’ feelings of being clear about personal values and being informed before and after their use of the web-based decision aid (one-tailed t-test for values clarity: *t_11_*=4.16; *P*<.001 and informed: *t_11_*=1.92; *P*=.04).

#### Integrated Analyses

Several changes made to V1 addressed comments that the decision aid was too complex and too dense. These comments were translated to one of the lowest usability scores across all versions (Table 3).

**Table 3:** Assessments of the successive versions of a web-based decision aid by mixed samples of older adults and health care professionals and impact of the decision aid on the perception of older adults of the clarity of their values and of being well-informed before (pre) and after (post) using the decision aid.

Outcome	Version 1 (n=6 HCP^a^)	Version 2 (n=3 OA^b^ and 1 HCP)	Version 3 (n=3 OA)	Version 4 (n=2 OA)	Version 5 (n=3 OA)	Version 6 (n=1 OA)	Total (n=12 OA and 7 HCP)
Willingness to use the decision aid—post^c^, mean (SD)	3 (1)	4 (0)	N/A^d^	N/A	N/A	N/A	N/A
System usability scale—post^e^, mean (SD)	56 (23)	65 (20)	77 (10)	56 (16)	75 (16)	55 (0)	N/A
Acceptability—post^f^, mean (SD)	76 (9)	84 (6)	92 (8)	82 (9)	86 (4)	73 (0)	N/A
Satisfaction—post^g^, mean (SD)	4 (1)	4 (1)	5 (0)	3 (1)	4 (1)	5 (0)	N/A
Values clarity (DCS^h^)—pre^i^, mean (SD)	N/A	64 (43)	64 (10)	38 (41)	75 (17)	50 (0)	61 (27)
Values clarity (DCS)—post^i^, mean (SD)	N/A	83 (8)	83 (14)	63 (30)	92 (8)	92 (0)	83 (16)
Pre-post mean values clarity (DCS) score increase	N/A	19	19	25	17	42	22
Informed (DCS)—pre^j^, mean (SD)	N/A	61 (54)	61 (24)	54 (30)	72 (27)	67 (0)	63 (30)
Informed (DCS)—post^j^, mean (SD)	N/A	69 (10)	92 (8)	79 (6)	92 (14)	67 (0)	82 (14)
Pre-post mean informed (DCS) score increase	N/A	8	31	25	20	0	19

^a^HCP: health care practitioner.

^b^OA: older adult.

^c^Scale from 1 to 5, with 5 indicating higher willingness. Answered only by HCP.

^d^N/A: not applicable, certain measures were used only with HCP or OA.

^e^Scale from 0 to 100, with 100 indicating higher usability. Answered by both OA and HCP.

^f^Proportion of people who find the web-based decision aid acceptable. Answered by both OA and HCP.

^g^Scale from 1 to 5, with 5 indicating higher satisfaction. Answered by both OA and HCP.

^h^DCS: decisional conflict scale.

^i^Scale ranging from 0 to 100, with 100 indicating higher clarity. Answered only by OA.

^j^Scale ranging from 0 to 100, with 100 indicating. Answered only by OA.

Comments on V2 came from 3 older adults and 1 HCP. Issues with content density were still prominent, as evidenced by one of the lowest increases in participants’ feelings of being informed before and after using the decision aid.

Changes in V3 increased the postdecisional comfort score on the *Informed* subscale from 61.1 to 91.7, which reflects the effort put into facilitating navigation. Version 3 had the highest acceptability (92%), usability (mean 76.7, SD 10.1), and satisfaction (mean 5.0, SD 0.0) scores, which confirmed the team’s efforts to layer the web-based decision aid to make it more manageable. The older adults still expressed misunderstanding of the values clarification exercise feedback page, which was the focus of the alterations in the next versions.

We made a final attempt at fixing the matrix table used on the VCE feedback page in V4. The older adults who evaluated this version presented relatively lower values clarity and perceptions of being informed before using the decision aid, and the qualitative comments indicated some frustration with the matrix table (the crux of the web-based decision aid). This seems to have influenced their assessments of the decision aid, as it scored the lowest in satisfaction and garnered one of the lowest usability scores.

In V5, we introduced a simpler values clarification exercise feedback format, which coincided with the highest posttest values for the *Informed* and *Values Clarity* measures, although the participants also had the highest scores in both of these subscales before using the decision aid. Nonetheless, these scores correspond to relatively high usability, acceptability, and satisfaction, and the comments appear to indicate that users understood this version of the feedback the best.

Version 6 introduced pictograms and a better classification of options to improve information recall, but it was tested with a single user only. The older adult that tested version 6 appreciated the usefulness of the pictograms. The highest increase in decisional comfort on the *Values Clarity* subscale was also measured in this round (mean_pre_ 50, mean_post_ 91.7), which suggests that the new feedback design achieved its goal. Usability (mean 55.0) and acceptability (mean 71.4) scores are, however, the lowest across all versions, but several comments made during data collection led us to believe that some of the SUS items may have been interpreted incorrectly. For example, to the SUS question “I think that I would need the support of a technical person to be able to use this system,” the sole participant that tested version 6 mentioned appreciating having access to technical resources in general, and thus gave a low usability score. These relatively low SUS scores also contrast with the user’s high level of satisfaction (mean 5.0) with the decision aid.

Users consistently demonstrated enthusiasm with regard to decision aid across all versions. HCPs sometimes expressed concerns about being able to integrate it into their practice, and older adults were not always sure that clinicians would care or have time to go through the whole process with them.

### Research Process

Throughout the data collection process, several observations were made. First, asking users to review a mock-up instead of a finished product requires much more involvement from the interviewer, which prevented us from observing real user interaction with the web-based decision aid. Second, having the users communicate their difficulties was not always easy because they generally felt that they understood the content even when it was clear to the interviewer that they had not. This highlights the importance of the evaluation approaches used in this project because observations of users interacting with the decision aid and teach-back allowed more issues with the web-based decision aid to be raised, compared with direct comments from users regarding their appreciation of it. Finally, spending time with community-based users and listening to them talk about their experiences and share their stories while navigating the web-based decision aid emphasized the wealth of information available from direct contact with users. Many modifications made to the web-based decision aid were first suggested by users, such as the addition of pictograms, the use of arrow buttons at the bottom of each page, and enlargement of the font size.

## Discussion

### Principal Findings

This study aimed to adjust the level of detail of a web-based decision aid and to apply features that best support evidence-informed and value-based decision making for older adults with MCI. Our main findings indicate that, to achieve this goal, the web-based decision aid needs to provide a guided decision-making process, the content needs to be simple but not simplistic, and the interface and navigation should be intuitive and user friendly.

### Guided Decision-Making Process

First, we found that a patient-centered web-based decision aid that helps adults >60 years with MCI to make a value-based decision performs better when it helps users clarify their priorities early on in the process. This allows for a tailored presentation of content that guides the user toward the most relevant options for their specific needs. Presenting fewer options at a time helps to avoid overwhelming the user with reading material about all available options, especially when there are several. Moreover, a study that was conducted with adults of various ages found that the first option presented in a decision aid was more likely to be chosen as a treatment option, especially when there were several options [49]. This suggests that it is important to present options in a way that reflects the user’s priorities, especially if their cognitive abilities may be impaired. It also coincides with the findings from a 2016 study that showed that a linear navigation had a higher success rate, lower performance time, better satisfaction ratings, and greater user preference than hypertextual navigation for elderly users [50]. In addition, it converged with studies showing that older people prefer having fewer options [13].

However, tailoring must be performed carefully to avoid removing essential information, as this could lead to bias in the decision-making process, increased decision conflict, and decreased knowledge [22]. More specifically, if a values clarification exercises is proposed to tailor information about options in a decision aid, then the aid should still allow access to information on all options as patients need sufficient knowledge of the options before being able to clarify their values [51].

Considering that web-based decision aids can also be used by HCPs and caregivers, in addition to older adults with MCI, they should remain flexible despite their general linearity, so people who prefer to either skip or read some sections exhaustively have the option to do so. In brief, the web-based decision aid should be directive rather than restrictive. This implies that, in the case of cognitively impaired people, the decision relies more heavily on the accuracy of the values clarification exercise, and therefore on the accurate pairing of each option with the priorities they are designed to meet.

### Simple, Not Simplistic

Next, our team resorted to information layering to simplify the design while retaining the level of detail to support informed, shared decision making. This helped provide a straightforward layout that allowed users to understand the decision aid’s structure. The text was also modified to use clear and comprehensible wording to retain only meaningful content and remove overwhelming details. These results converge with the findings of Peters [12] that *less is more* when it comes to making informed and high-quality decisions, especially in people with lower numeracy skills. Considering that people with MCI often have episodic memory impairments [52], it is important that the information be concise, meaningful, and easy to remember. Our results indicate that the pictograms are a useful strategy to help users remember the options available and, in the process, summarize the information into a visual reminder. These findings converge with those found in literature reviews, indicating that pictograms are good for, among other things, increasing visual attention and recall [53,54].

In our streamlining efforts, we had to be careful about oversimplifying the decision aid’s content. Although users might find it easier for someone else to make the decision for them, studies show that informing them of the benefits and harms supports better outcomes [55]. Therefore, even if probabilities are hard to understand for most users, they are the very core of the decision aid, and we tried several approaches to make them clearer. The scientific literature reviews several ways of presenting risk probabilities (benefits and harms). Most agree that percentages and natural frequencies using a uniform denominator, as was done in the final version of the web-based decision aid, are the most effective way to present risks [56,57]. It has also been found that when visual aids such as icon arrays and bar graphs are added, comprehension—and retention—of the numbers increases significantly [57,58]. Nevertheless, other findings suggest that people with low graph literacy understand risks presented in numbers better, whereas people with high graphical literacy skills better understand those presented in graph form more readily [59]. It would be interesting to test a web-based decision aid that offers a graphical display of the risks in a layer for people seeking to learn more. Having said that, when an analysis was made of the participants’ perceptions of being informed and clear about their personal values before and after using the web-based decision aid, it showed that their comfort with decision making generally improved with the use of the tool.

### Intuitive and User Friendly

Finally, we conclude from our findings that users require a seamless experience when navigating the web-based decision aid to avoid any distraction and frustration with the decision-making process. The SUS scores that we measured throughout the rounds suggest that we achieved *good* usability at best, according to Sauro’s reported average score of 68 across studies [44], despite several rounds of user testing. Some comments also suggest that the SUS items were not clearly understood, which might warrant validation in this population. These findings converge with those of Malinowsky et al [60], who found that older adults with MCI experience more difficulty using technology than those without any cognitive problems. Although further testing might help us reach greater usability, this emphasizes the importance of testing tools with the intended demographic during the design phase, to avoid creating decision aids that hinder the decision-making process instead of helping it. Our results show improved usability when the interface displays clear components that require little interaction (eg, checkboxes instead of drop-down menus) when page jumping is minimized (eg, pop-ups and information tips) and when all page content fits on a single screen, thereby eliminating the need to scroll down. This last observation converges with the results of another study on the implementation of a mobile health app, showing the value of content that is *at one’s fingertips* [45]. This is also consistent with the guidelines of the United States Department of Health and Human Services [61], which are based on Morville and Rosenfeld’s book [62] and which state that the interface should stay simple, with clear instructions and a page structure that is clear in its purpose [61].

### Limitations of the Study

One of the limitations of the study is that the web-based decision aid shown at each round to the participants was a mock-up. This approach allowed us to minimize costs, to avoid programming several test versions of the decision aid, and is a standard approach used in website design. However, the mock-up functionality was limited and caused some unintended glitches. It is difficult to estimate the extent to which this affected the participants’ understanding of the web-based decision aid in general; however, the mock-up clearly impaired understanding of the values clarification exercise. In the mock-up, we had to predetermine values clarification exercise priorities to avoid unnecessary design work at an early stage. Knowing that older adults with MCI present deficits in their executive functions and working memory [63], it is possible that the task of planning for a fictitious case was even more difficult for them, perhaps even impossible for some. Many of the participants with MCI were confused or even frustrated at not being able to put their real priorities in the values clarification exercise. This type of reaction was also found in another study to build a web-based shared decision-making tool with older adults living with neurocognitive disorders [64]. We recommend using a fully functional values clarification exercise when testing prototypes for this type of participant.

This work is also limited by the small sample sizes of HCPs and older adults with MCI. The vulnerability of the population made it hard to recruit subjects with the help of HCPs, as they wanted to protect them from the hassles of a research project. Consequently, we had to rely on alternative strategies to recruit older adults with MCI. We could not, therefore, diversify the participants who helped at each evaluation round, for example, for levels of MCI, educational background, and technology skills. Further research projects could lead to qualitative interviews with a smaller number of participants while simultaneously asking a larger group to test the web-based decision aid. This would allow for the analysis of a larger amount of quantitative data while retaining the richness of the qualitative data gathered during the interviews.

Potential limitations of the quantitative data collected should also be clarified. The DCS that we used to help us tailor the decision aid measures only feelings about the decision, not the degree to which the values presented coincided with the individuals’ actual values. In addition, given that participants were face to face with their interviewers, some may have answered in a way they felt was expected of them (social desirability bias).

### Conclusions

Overall, we can say that we have successfully identified and applied features that best support values clarification and understanding of evidence in older adults with MCI and have adjusted the level of detail of a web-based decision aid for individuals with MCI, although there is still room for improvement. For example, we designed this interactive decision aid for tablets and computers, whereas internet access is increasingly used via mobile devices.

In conclusion, this study resulted in a promising web-based decision aid. Further ongoing research will allow its implementation to be tested in clinical settings. This decision aid will be useful in supporting health workers who regularly interact with older adults, such as nurses, physicians, geriatricians, social workers, psychologists, community pharmacists, or volunteers from community organizations. This decision aid will allow HCPs to meaningfully engage older adults in the decision-making process, so that patient values are prioritized. It will also be useful in informing older adults with MCI of the options available to improve their cognitive abilities, allowing them to feel a greater sense of control over their condition.

Our findings support three recommendations to create web-based decision aids for older adults with MCI:

1. Although we recognize that some evidence supports understanding options before completing a values clarification exercise [5,51], our research suggests that the website should guide users in completing a values clarification exercise before presenting any options, allowing for tailoring of the presentation of options to the users’ priorities, while also allowing flexibility for those users who prefer jumping from one section to the other in no specific order.
2. Content should be simple, but not simplistic, notably by using information layering, lay language, and pictograms to represent each option.
3. The interface should be intuitive and user friendly, for example, by preferring check marks to drop-down menus, using pop-up windows and information tips, and by limiting the height of the window to avoid having to scroll down.

We also recommend that decision-aid developers consult users early on in the design process and combine observations of user interactions with the decision aid with a teach-back approach, to collect rich information from the get-go, and to ensure that they design an understandable and usable web-based decision aid for this more vulnerable population.

## Appendix

Multimedia Appendix 1 Introduction page of the web-based decision aid, as tested in prototype version 1. Warning: this is a preliminary version, and the data are not final.

Multimedia Appendix 2 Introduction page of the web-based decision aid, as seen in the final version.

Multimedia Appendix 3 Definition appearing as an info-tip when the user hovers the cursor over a word, in the final version of the web-based decision aid.

Multimedia Appendix 4 Decision page in the initial prototype.

Multimedia Appendix 5 Decision page in the final version.

## Abbreviations

DCS: decisional conflict scale
GRADE: grading of recommendations assessment, development, and evaluation
HCP: health care professionals
MCI: mild cognitive impairment
SUS: system usability scale
