# Supplementary figures and images for: User-Centered Design and Evaluation of a Web-Based Decision Aid for Older Adults Living With Mild Cognitive Impairment and Their Health Care Providers: Mixed Methods Study
Source: J Med Internet Res. 2020 Aug 19;22(8):e17406. doi: 10.2196/17406 (PMC7468645; doi:10.2196/17406)

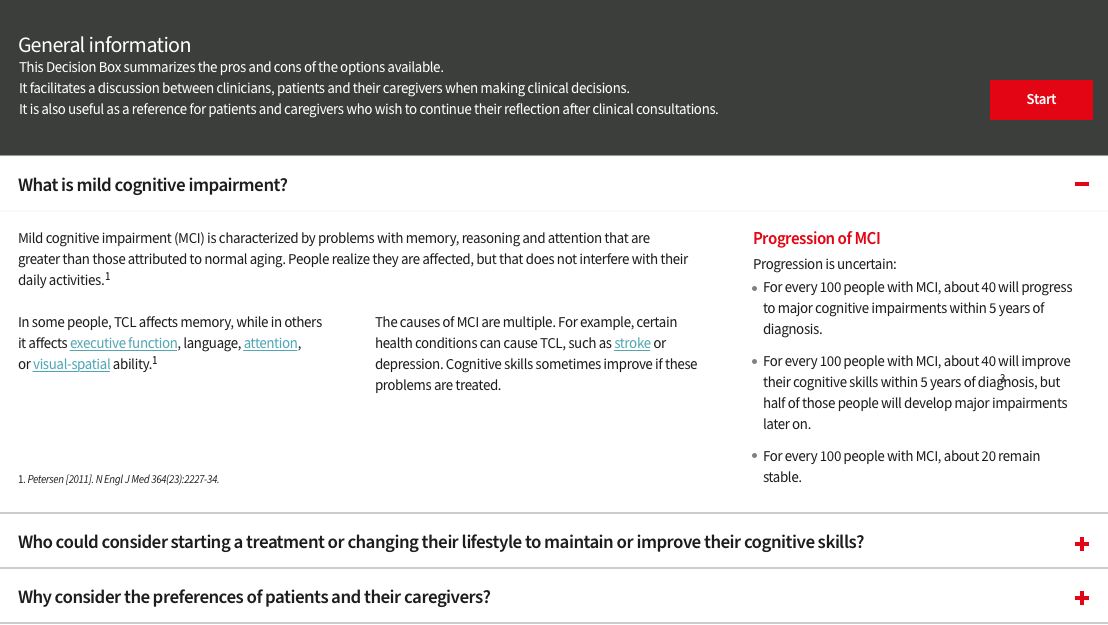

Supplement: Multimedia Appendix 1 [file jmir_v22i8e17406_app1.png]

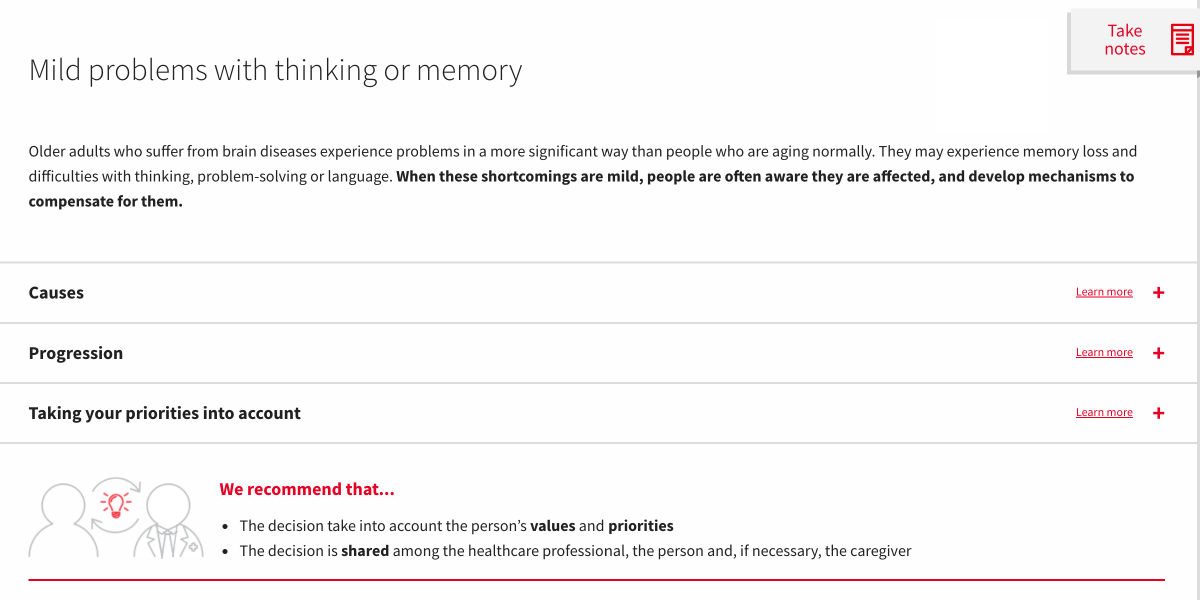

Supplement: Multimedia Appendix 2 [file jmir_v22i8e17406_app2.png]

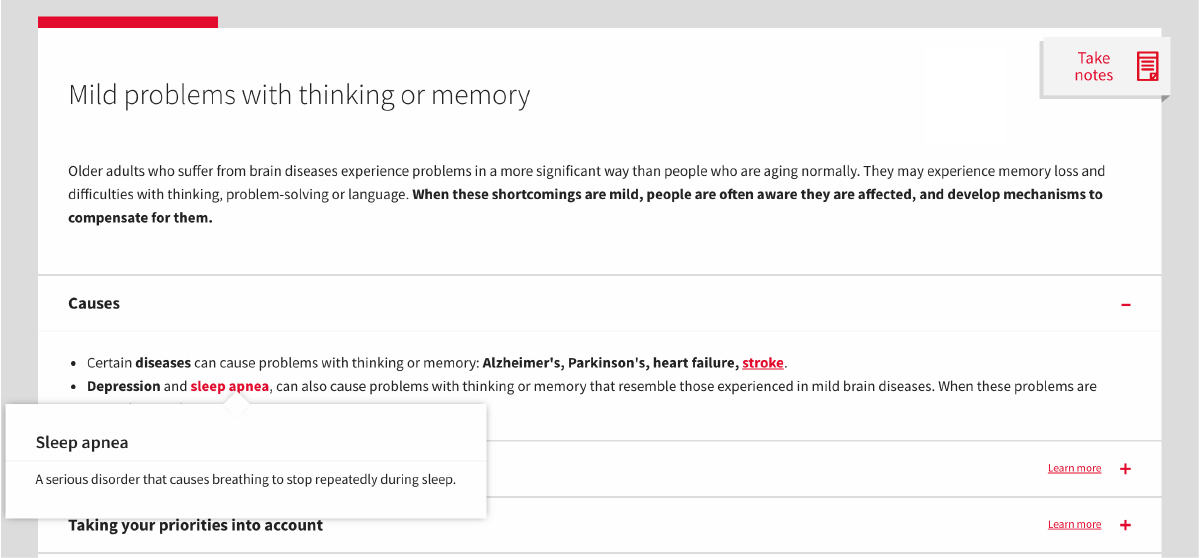

Supplement: Multimedia Appendix 3 [file jmir_v22i8e17406_app3.png]

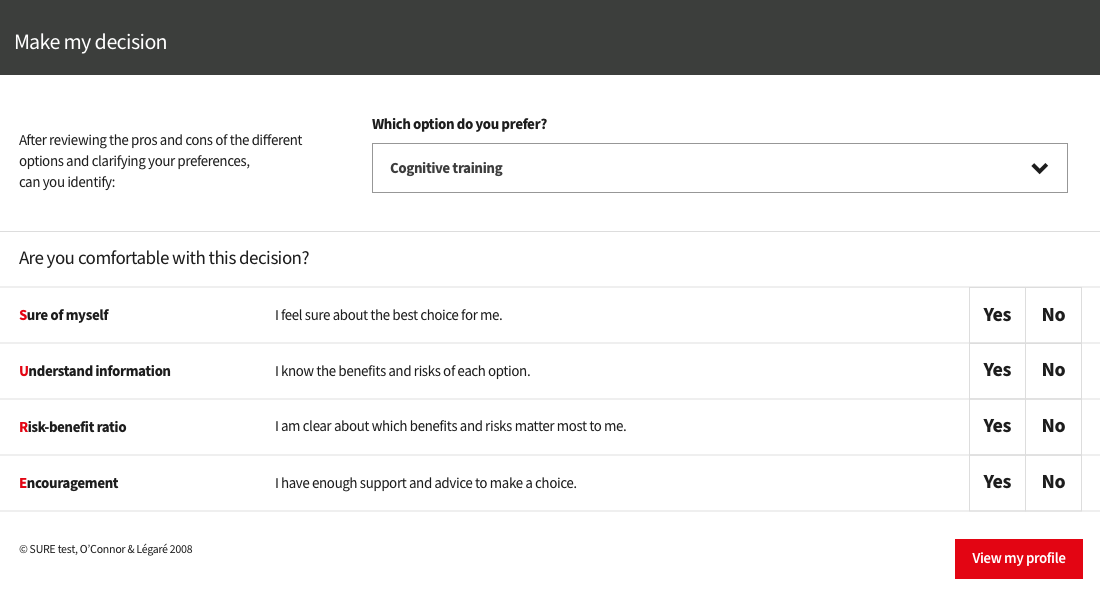

Supplement: Multimedia Appendix 4 [file jmir_v22i8e17406_app4.png]

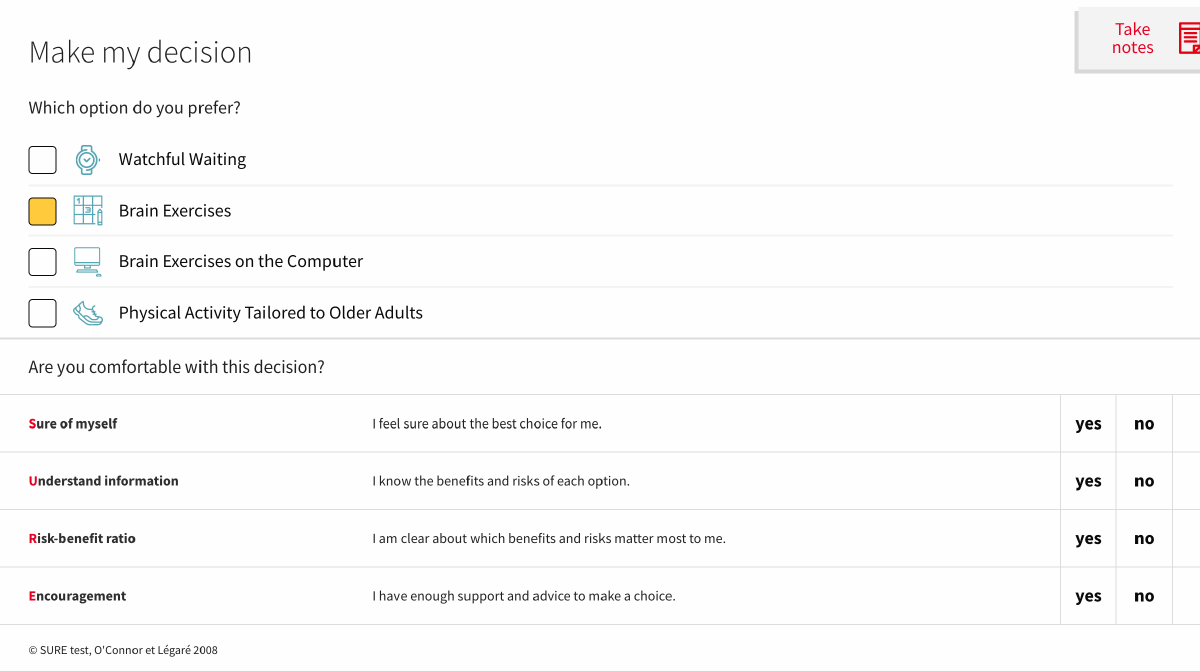

Supplement: Multimedia Appendix 5 [file jmir_v22i8e17406_app5.png]
